# A game-theoretic framework for multimodal information utilization under heterogeneous processing environments in neuroscience and perception science

**DOI:** 10.3389/fnins.2026.1829021

**Published:** 2026-05-01

**Authors:** Zhanhong Cui, Kai Li

**Affiliations:** School of Business Administration, Northeastern University, Shenyang, China

**Keywords:** game-theoretic framework, heterogeneous environments, information asymmetry, multimodal information utilization, neuroscience, perception science

## Abstract

Multimodal data integration is increasingly central to neuroscience and perception science, where heterogeneous signals such as behavioral responses, sensory inputs, electrophysiological recordings, neuroimaging measurements, and computational representations must be jointly interpreted. Based on the realistic background, there is a core theoretical problem that needs further research: under what heterogeneous processing conditions does enhanced multimodal information utilization produce meaningful gains, when does it become strategically necessary, and when does it generate only limited benefits relative to its cost? To clarify this core problem, this study develops a conceptual game-theoretic framework in which information utilization is treated not as a universally beneficial technical upgrade, but as a conditional strategic choice shaped by signal heterogeneity, information asymmetry, integration cost, and differential decision influence across actors. Within this framework, we compare three endogenous strategic profiles—no enhanced information utilization, unilateral information enhancement, and bilateral information enhancement—across multiple heterogeneous environments. The analysis results show that the value of multimodal information utilization is fundamentally environment-dependent. In highly homogeneous environments, additional information processing yields little marginal benefit and is therefore not sustained in equilibrium. In moderately heterogeneous environments, however, multimodal information utilization emerges as a strategically necessary response because it reduces mismatch, improves alignment, and stabilizes decision outcomes. In more asymmetric environments, stronger decision agents capture a disproportionate share of the gains from enhanced information utilization and increasingly rely on differentiated strategic responses, whereas weaker agents adopt more defensive and uniform strategies. In highly dominated environments, the marginal value of additional information utilization declines again because structural dominance itself already secures most attainable advantages. These findings contribute to multimodal neuroscience and perception science by clarifying that the consequences of information utilization depend not only on fusion efficiency, but also on environmental structure, asymmetry, and the distribution of strategic power.

## 1 Introduction

Multimodal data processing has become an increasingly important methodological direction in neuroscience and perception science. Perceptual and cognitive processes are rarely expressed through a single signal alone; rather, they are jointly reflected in behavioral responses, sensory inputs, electrophysiological activity, neuroimaging measurements, and computational representations. Compared with single-modality analysis, multimodal integration offers a richer basis for characterizing how the brain encodes, aligns, and interprets information across time and space. Recent studies published since 2023 have further shown that the integration of EEG, MEG, fMRI, fNIRS, behavioral measures, and multimodal machine learning is becoming central to the investigation of neural dynamics, multisensory perception, cognition, and clinically relevant decision support (Porcaro et al., 2023; Ghosh et al., 2024; Roman-Gonzalez, 2024; Baghdadi et al., 2025; Liu and Wang, 2025).

Yet, despite this progress, an important theoretical problem remains insufficiently formulated. Existing multimodal studies usually emphasize the technical benefits of adding information sources, but they less often explain why multimodal information utilization is advantageous in some environments, only conditionally effective in others, and potentially redundant in still others. This gap matters because multimodal systems are not deployed in a vacuum. In neuroscience and perception science, different modalities vary substantially in temporal resolution, spatial precision, semantic granularity, uncertainty, missingness, and acquisition cost. As a result, the same information-utilization strategy may improve alignment and interpretability in one setting while introducing redundancy, instability, or excessive integration burden in another (Brahim et al., 2011; Taylor, 2012; Zhang and Katona, 2012; Amatriain, 2013; Haans et al., 2013; Qu and Wang, 2021).

Accordingly, the central concern of this paper is not whether multimodal information is useful in an absolute sense, but under what conditions it creates meaningful strategic and system-level value. This issue is directly relevant to multimodal neuroscience and perception science for at least three reasons. First, multimodal studies routinely involve heterogeneous evidence sources whose relative value is uneven across tasks. Second, multimodal inference often depends on how asymmetries in information quality, reliability, or influence are handled rather than merely on the number of modalities included. Third, real multimodal systems involve nontrivial trade-offs among alignment quality, integration cost, interpretability, and downstream decision performance (Rochet and Tirole, 2003, 2006; Armstrong, 2006; Armstrong and Wright, 2007; Wright, 2010; Evans, 2011; Hermalin and Katz, 2016; Patro et al., 2020).

To address these issues, this paper develops a game-theoretic framework for multimodal information utilization under heterogeneous processing environments. A strategic framework is needed because multimodal information utilization is not simply a passive technical operation; it alters the incentives, responses, and payoff structure of the participating agents. Concepts such as asymmetry, cost, and strategic response are therefore directly relevant. Information asymmetry captures the fact that different actors or modalities may not contribute equally to inference. Integration cost captures the reality that richer multimodal processing requires additional synchronization, transformation, calibration, and system complexity. Strategic response captures the fact that once one side enhances its information utilization, other sides may respond defensively, symmetrically, or differentially. For these reasons, a game-theoretic perspective provides a useful analytical language for understanding why the value of multimodal information utilization is conditional rather than universal (Bergemann and Bonatti, 2011; Esteves and Resende, 2016).

More specifically, this study addresses the following three research questions:

1. Under what heterogeneous processing conditions does enhanced multimodal information utilization generate meaningful gains in matching quality, decision stability, and system-level outcomes?
2. When asymmetry exists across actors or information sources, does equilibrium behavior favor symmetric information utilization or selective and differentiated utilization?
3. How do different information-utilization profiles reshape the distribution of gains among consumers, platforms, and merchants across heterogeneous environments?

To answer these questions, we distinguish several heterogeneous environments that differ in structural asymmetry, bargaining conditions, baseline differentiation, and information-processing cost. Within each environment, we compare three endogenous strategic profiles: no enhanced information utilization, unilateral information enhancement, and bilateral information enhancement. This design makes it possible to identify not only whether additional multimodal information is adopted, but also how its strategic role changes across environmental regimes.

The contribution of this study is threefold. First, it provides a unified conceptual framework linking multimodal information utilization, strategic response, and system-level outcomes across heterogeneous environments rather than within a single application scenario. Second, it advances current discussions in multimodal neuroscience and perception science by showing that the consequences of information utilization depend on asymmetry, cost, and environmental structure, not only on technical fusion capability. Third, it offers a theoretically interpretable explanation for why multimodal information utilization may be irrelevant in highly homogeneous environments, strategically necessary in moderately heterogeneous ones, asymmetry-reinforcing in uneven environments, and redundant again in highly dominated settings.

The remainder of the paper is organized as follows. Section 2 reviews the related literature on multimodal data processing, information asymmetry, and strategic decision-making in heterogeneous environments. Section 3 presents the analytical framework, including the processing environment, information-utilization strategies, utility structure, cost setting, and equilibrium concept. Section 4 reports the comparative results across different environments and provides visual summaries of the strategic and welfare implications. Section 5 concludes the paper by synthesizing the main theoretical contributions, discussing limitations, and outlining future research directions.

## 2 Literature review

### 2.1 Multimodal data integration in neuroscience and perception science

A major development in recent neuroscience and perception science is the transition from single-modality observation to multimodal data integration. Perception and cognition are rarely reflected in only one form of evidence; instead, behavioral responses, electrophysiological activity, neuroimaging signals, physiological measurements, and computational representations often capture complementary but heterogeneous aspects of the same underlying process. Recent editorials, reviews, and application studies have emphasized that multimodal processing is becoming a central methodological paradigm for studying perception, cognition, and brain dynamics, particularly when neural, behavioral, and sensory information must be interpreted jointly rather than separately (Fan et al., 2023; Roman-Gonzalez, 2024; Ghosh et al., 2024; Wang et al., 2025).

This trend is especially visible in studies combining modalities with distinct strengths. EEG and related electrophysiological signals provide high temporal resolution, whereas fMRI and fNIRS offer stronger spatial characterization; behavioral and perceptual responses contribute task-level interpretability, while multimodal machine learning helps bridge these heterogeneous representations. Recent work has shown that such combinations can improve the characterization of neural dynamics, perceptual decoding, and clinically relevant inference when complementary information is effectively integrated (Phadikar et al., 2025; Yang et al., 2025; Bhattacharya et al., 2025; Jiricek et al., 2025).

In parallel, the field has seen rapid progress in multimodal datasets and benchmark resources. Newly released datasets provide aligned EEG, MRI, behavioral, auditory, semantic, and imagery-related signals for studying perceptual normalization, semantic decoding, and multimodal neural representation. These resources are important because they improve reproducibility, facilitate method comparison, and support more realistic modeling of perceptual and cognitive tasks (Liu and Wang, 2025; Chen et al., 2025; Rybář et al., 2025; Shi et al., 2025).

Despite these advances, recent studies also make clear that multimodal integration is not uniformly beneficial. Additional modalities do not automatically improve inference merely because more signals are available. Rather, their value depends on whether the modalities are sufficiently complementary, whether they can be aligned meaningfully, and whether their informational contribution outweighs the increased burden of acquisition, preprocessing, and model integration. This observation motivates the need for a more general conceptual framework for understanding when multimodal information utilization is advantageous and when its marginal benefit remains limited (Fan et al., 2023; Ghosh et al., 2024; Roman-Gonzalez, 2024).

### 2.2 Heterogeneity, cross-modal alignment, and uncertainty

A second major theme in the recent literature concerns the technical and theoretical challenges created by heterogeneity across modalities. In neuroscience and perception science, different signals often vary substantially in temporal scale, spatial precision, semantic granularity, noise structure, and susceptibility to missingness. EEG may capture fast neural dynamics but offer relatively weak spatial localization; fMRI can identify distributed activation patterns but blur temporal information; peripheral physiological signals may provide useful complementary evidence but are often indirect and noisy. Accordingly, recent studies emphasize that the principal challenge is not simply collecting more data sources, but integrating them in a way that preserves complementary structure while controlling mismatch and uncertainty (Ghosh et al., 2024; Phadikar et al., 2025; Yang et al., 2025).

Recent work increasingly treats cross-modal alignment as a core methodological problem. Studies on simultaneous EEG–fMRI fusion, multimodal semantic decoding, and multimodal neuroimaging resources show that successful integration depends on resolving asynchronous structure, representation mismatch, and context-dependent correspondence across sources. In this sense, multimodal fusion is not only a feature-combination problem, but also a problem of determining which sources are commensurable, which are complementary, and which should be weighted differently in different tasks (Chen et al., 2025; Liu and Wang, 2025; Rybář et al., 2025; Jiricek et al., 2025).

Uncertainty has likewise become a recurring concern in multimodal studies. When modalities differ in quality or reliability, naïve fusion may propagate noise rather than improve inference. This is especially evident in physiological state recognition, affective computing, and clinical monitoring tasks, where multimodal systems often succeed only when signal variability, modality-specific reliability, and uncertainty are explicitly modeled rather than ignored (Fu et al., 2023; Wang et al., 2025; Ebato et al., 2025; Wei et al., 2025).

Related concerns are also visible in rehabilitation, neurological assessment, and multimodal classification settings. Recent work on hybrid EEG–fNIRS datasets, multimodal monitoring, and integrated imaging-analysis pipelines indicates that heterogeneity should be treated as a structural property of multimodal evidence rather than as a simple nuisance variable. Taken together, these findings suggest that multimodal information utilization should be understood as a conditional process shaped by alignment quality, uncertainty, and compatibility across sources (Shi et al., 2025; Wei et al., 2025; Bhattacharya et al., 2025; Wen et al., 2025).

### 2.3 Decision-oriented multimodal information utilization and remaining gaps

A third stream of recent literature most directly related to this study concerns how multimodal information utilization influences downstream decision structure. In applied neuroscience and perception science, multimodal processing is rarely valuable in itself; instead, its significance lies in whether it improves diagnosis, decoding, monitoring, classification, or theoretical interpretation. Recent work on brain–computer interfaces, multimodal clinical modeling, and neurological disorder analysis shows that additional modalities can improve system performance when they provide genuinely complementary evidence, but they may also introduce redundancy, instability, or interpretability problems when their contribution is weakly aligned or marginal (Lee et al., 2002; Khan et al., 2024; Li et al., 2025; Teng et al., 2025; Jin et al., 2025).

This issue becomes especially important in asymmetric environments, where different modalities do not contribute equally to inference. Some recent studies suggest that one modality may function as the dominant source of evidence, whereas others play supplementary or corrective roles. In such cases, selective or asymmetric information utilization may be more effective than treating all modalities as equally informative. This pattern has been observed in multimodal fusion for Parkinson's disease, EEG–fMRI integration, and brain–computer interface classification, where gains depend not only on the number of modalities but also on how their relative influence is structured (Li et al., 2010; Phadikar et al., 2025; Li et al., 2025; Teng et al., 2025; Zhi et al., 2026).

Integration cost is another underdeveloped issue in the recent literature. Additional modalities often require synchronization, preprocessing, feature transformation, uncertainty calibration, and more complex model architectures. They may also reduce interpretability or impose practical burdens in acquisition and deployment. For this reason, recent ethical and methodological discussions increasingly argue that multimodal systems should be evaluated not only by predictive performance, but also by reliability, transparency, scalability, and responsible use in real applications (Hurley et al., 2024; Roman-Gonzalez, 2024; Wei et al., 2025; Li et al., 2026).

Despite substantial recent progress, the literature still lacks a unified conceptual explanation of how multimodal information utilization changes across heterogeneous environments. Existing studies often focus on a particular modality pair, one clinical condition, or one fusion architecture, while broader theoretical questions remain insufficiently addressed. In particular, three issues remain open. First, under what heterogeneous processing conditions does additional multimodal information produce meaningful gains? Second, when asymmetry exists across modalities, should information be utilized symmetrically or selectively? Third, how do different information-utilization profiles affect decision quality, stability, and system-level effectiveness? These unresolved questions are highly relevant to current neuroscience and perception science, where multimodal methods are expanding rapidly but still face major challenges in alignment, uncertainty management, and robust decision support. Against this background, the present study develops a conceptual framework to analyze multimodal information utilization, decision strategy, and system-level outcomes across heterogeneous processing environments.

## 3 Methodology

To improve conceptual clarity before presenting the formal derivation, Table 1 summarizes the major variables and parameters used in the model, together with their definitions and expected analytical roles. This table is intended to make the structure of the model more transparent and to clarify how each variable contributes to the strategic logic of multimodal information utilization under heterogeneous environments.

**Table 1:** Summary of major variables and parameters in the model.

Symbol	Definition	Expected role in the model
γ	Relative platform asymmetry parameter	Captures the degree to which one platform has stronger traffic allocation, visibility, or competitive influence. Higher asymmetry tends to widen strategic and payoff differences.
δ	Merchant bargaining asymmetry parameter	Measures the relative strength of merchants in resisting platform extraction. It affects effective commissions, bargaining outcomes, and the outside option.
*t*	Baseline mismatch intensity	Represents the initial degree of mismatch between consumer preferences and platform offerings before enhanced information utilization.
*t*_*i*_	Information-utilization intensity of platform *i*	Measures how intensively platform *i* processes user information to reduce mismatch and improve matching quality.
A	Composite structural asymmetry index	Aggregates the joint effect of platform asymmetry and bargaining asymmetry; used to characterize how unbalanced the environment is.
*x*_0_	Outside-option share	Captures the portion of demand not covered by either platform. It expands when asymmetry or excessive prices reduce market attractiveness.

This section develops the analytical framework used to examine how information-utilization strategies evolve across heterogeneous competitive environments. Consistent with the preceding literature review, the model starts from the premise that information utilization is not universally beneficial. Its strategic value depends on the joint effect of market asymmetry, bargaining conditions, product differentiation, and information-processing cost. Accordingly, the methodological purpose of this section is not simply to construct a game-theoretic model, but to establish a unified analytical system in which information utilization, market response, and welfare consequences can be compared across multiple structural environments.

**Figure 1:**
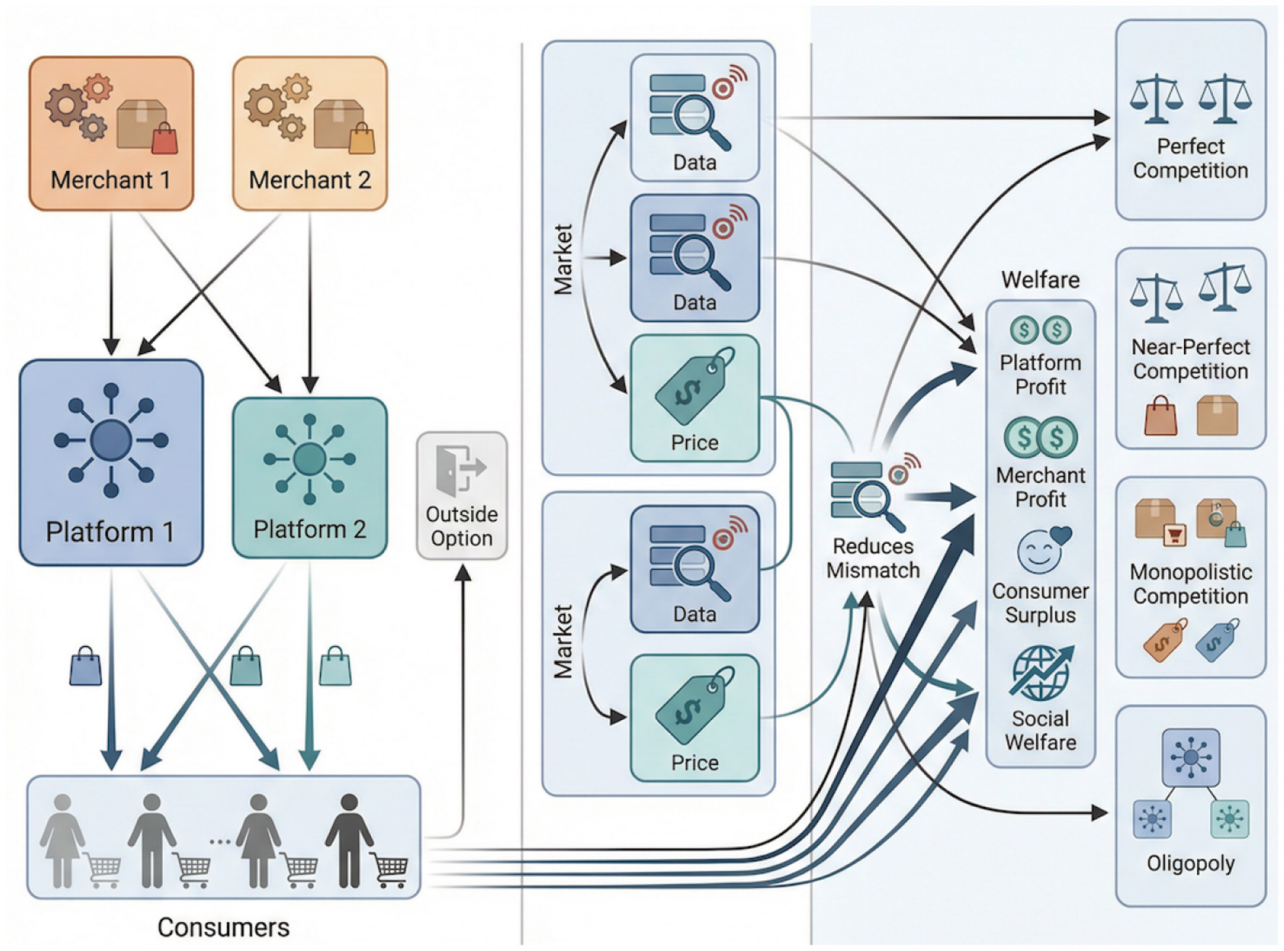
Overview of the analytical framework linking environmental heterogeneity, information-utilization strategies, market allocation, and welfare outcomes.

Figure 1 summarizes the logic of the framework. The model is organized around three interconnected layers. The first layer is the *environmental layer*, which characterizes platform asymmetry, merchant bargaining conditions, and product differentiation. The second layer is the *strategic layer*, in which platforms determine information-utilization intensity and commission policies. The third layer is the *outcome layer*, where these strategic choices reshape consumer matching, market allocation, profit extraction, and welfare distribution. This layered structure allows the same information mechanism to be evaluated under different competitive regimes rather than only in a single market configuration.

### 3.1 Modeling objective and sequential structure

This sequential structure is theoretically important because it mirrors a decision-oriented multimodal environment in which information processing changes not only direct matching performance, but also the strategic responses of downstream participants. In other words, multimodal information utilization is modeled as a mechanism that reshapes the full decision chain rather than merely as a static technical improvement. The analytical objective is to characterize how platforms select information-utilization strategies when they face heterogeneous competitive conditions and different cost-benefit trade-offs. To do so, the model incorporates four structural dimensions into a unified system (Dobson et al., 2001; Gao, 2011; Pan and Yang, 2014; Martens, 2021):


$$\begin{array}{r} ℰ=\{\underset{\text{platform asymmetry}}{\underset{︸}{\gamma }},\underset{\text{bargaining asymmetry}}{\underset{︸}{\delta }},\underset{\text{baseline differentiation}}{\underset{︸}{t}},\\ \underset{\text{information cost parameters}}{\underset{︸}{\lambda ,\mu }}\}. \end{array}$$ (1)

According to Equation 1, the game unfolds sequentially. In Stage 1, platforms choose commission parameters and information-utilization intensities. In Stage 2, upstream merchants respond by setting prices. In Stage 3, consumers allocate demand across the two platforms and the outside option. The equilibrium is solved by backward induction. This sequence is important because it allows information utilization to affect outcomes through two channels simultaneously: first, by changing effective matching quality; second, by changing the way upstream agents respond through pricing.

### 3.2 Market structure and asymmetry representation

Consider a market with two competing platforms, indexed by *i* ∈ {1, 2}, and two upstream merchants, indexed by *j* ∈ {1, 2}. Each merchant supplies one unit of a horizontally differentiated product through one platform, and each consumer purchases at most one unit.

To capture both horizontal and vertical asymmetry in a parsimonious but structurally interpretable way, define


$$\begin{array}{l} \Gamma =\left(\gamma ,1-\gamma \right),\;\Delta =\left(\delta ,1-\delta \right),\;\gamma ,\delta \in \left(0,1\right), \end{array}$$ (2)

According to Equation 2, where γ measures relative platform strength and δ measures relative merchant bargaining strength. A larger γ indicates stronger platform-side influence in traffic allocation, visibility, and competitive leverage, whereas a larger δ implies stronger merchant-side ability to resist vertical extraction.

Rather than viewing these parameters independently, the Equation 3 treats them as jointly shaping the effective competitive environment. Specifically, define the composite structural asymmetry index


$$\begin{array}{l} A=\omega_{\gamma }{\left(2\gamma -1\right)}^{2}+\omega_{\delta }{\left(2\delta -1\right)}^{2},\;\omega_{\gamma },\omega_{\delta }>0, \end{array}$$ (3)

which increases as the market deviates from symmetry in either horizontal competition or vertical bargaining. This index is used below to formalize outside-option expansion and strategic pressure under asymmetric environments. The asymmetry specification is deliberately parsimonious. Rather than introducing many separate imbalance indicators, the model uses γ and δ to represent the two most important structural dimensions: horizontal platform-side asymmetry and vertical bargaining asymmetry. This choice improves interpretability and allows the comparative results to be traced back to economically meaningful structural differences.

### 3.3 Information utilization and matching improvement

In Equation 4, the core strategic variable is the extent to which each platform utilizes consumer information. Let *t*_*i*_ ≥ 0 denote the information-utilization intensity of Platform *i*. This variable captures the ability of the platform to process user data, infer heterogeneous preferences, and reduce effective mismatch through recommendation, targeting, and traffic allocation.

Unlike a binary adoption setting, the present model treats information utilization as a continuous strategic instrument. Let *t*>0 denote the baseline mismatch intensity in the absence of recommendation. Then


$$\begin{array}{l} 0\leq t_{i}<t,\;i=1,2, \end{array}$$ (4)

so that recommendation mitigates but does not completely remove preference frictions.

For later comparative analysis, the main strategic profiles can be represented compactly as Equation 5


$$\begin{array}{l} S=\left\{\text{NN}:\left(0,0\right),\text{DN}:\left(t_{1},0\right)\text{ or }\left(0,t_{2}\right),\text{DD}:\left(t_{1},t_{2}\right)\right\},\\ \;t_{1},t_{2}>0\text{ under DD}. \end{array}$$ (5)

These profiles are not externally imposed cases, but endogenous strategic outcomes that may arise under different parameter regions.

### 3.4 Consumer utility with endogenous matching frictions

Consumers are uniformly distributed on the interval [0, 1]. Platform 1 is located at 0 and Platform 2 is located at 1. Let *x* denote the position of a representative consumer. The utility from transacting through Platform *i* is assumed to depend jointly on product price, merchant-side quality, information-enhanced matching, and residual mismatch frictions.

We define indirect utility as Equation 6


$$\begin{array}{l} U_{i}\left(x\right)=V-\alpha_{i}p_{i}+\beta_{i}e_{i}+\vartheta_{i}t_{i}-\eta_{i}d_{i}\left(x\right)-\frac{\chi_{i}}{2}d_{i}{\left(x\right)}^{2},\;i=1,2, \end{array}$$ (6)

where *V* is the common base value of participation, *p*_*i*_ is the price charged to consumers, *e*_*i*_ is the merchant-side service or network benefit, and *d*_*i*_(*x*) is effective mismatch. Relative to the simple linear utility form, Equation 6 introduces two features. First, the term ϑ_*i*_*t*_*i*_ captures the direct informational value of recommendation. Second, the quadratic friction term $\frac{\chi_{i}}{2}d_{i}{\left(x\right)}^{2}$ allows mismatch costs to rise nonlinearly when recommendation fails to sufficiently align consumers and products (Hann et al., 2008; Tang et al., 2008; Campbell et al., 2015; De Corniere and De Nijs, 2016; Shy and Stenbacka, 2016; Ichihashi, 2020; Wei et al., 2023; Zhao and Ding, 2023).

To embed environmental asymmetry directly into consumer evaluation, the sensitivity parameters are written as Equations 7–9


$$\begin{array}{l} \alpha_{1}=\frac{1-\gamma }{\delta }+\psi {\left(2\gamma -1\right)}^{2},\;\alpha_{2}=\frac{\gamma }{1-\delta }+\psi {\left(2\gamma -1\right)}^{2}, \end{array}$$ (7)

$$\begin{array}{l} \beta_{1}=\frac{\gamma }{1-\delta }+\phi \delta ,\;\beta_{2}=\frac{1-\gamma }{\delta }+\phi \left(1-\delta \right), \end{array}$$ (8)

$$\begin{array}{l} \eta_{1}=\frac{\xi_{1}\left(1-\gamma \right)}{\delta },\;\eta_{2}=\frac{\xi_{2}\gamma }{1-\delta },\;\vartheta_{i},\chi_{i},\psi ,\phi ,\xi_{i}>0. \end{array}$$ (9)

This specification implies that market asymmetry does not affect only prices or bargaining outcomes directly; it also changes how consumers translate platform-side and merchant-side differences into utility. Conceptually, *t*_*i*_ should be understood as a reduced-form measure of multimodal information utilization capability. It may reflect better user profiling, stronger multimodal representation learning, more accurate preference inference, or more effective recommendation and allocation mechanisms. Modeling *t*_*i*_ as a continuous variable rather than a binary choice allows the framework to distinguish not only whether information utilization is adopted, but also how intensively it is deployed under different environments.

### 3.5 Effective mismatch, outside option, and demand allocation

Information utilization modifies the mismatch structure faced by consumers. Let the effective mismatch functions be


$$\begin{array}{l} d_{1}\left(x\right)=\frac{\left(t-t_{1}\right)x}{1+\kappa t_{1}},\;d_{2}\left(x\right)=\frac{\left(t-t_{2}\right)\left(1-x-x_{0}\right)}{1+\kappa t_{2}},\;\kappa >0, \end{array}$$ (10)

so that information utilization reduces mismatch not only linearly through (*t* − *t*_*i*_), but also through a diminishing-friction adjustment captured by the denominator 1+κ*t*_*i*_. This reflects the idea that information processing has a nonlinear effect on the effective quality of matching.

The outside-option share is modeled as a function of structural asymmetry:


$$\begin{array}{l} x_{0}=\frac{1}{2}{\left(1-2\gamma \right)}^{2}+\frac{\tau }{2}{\left(1-2\delta \right)}^{2}+\zeta {\left[\frac{p_{1}+p_{2}}{2}-V\right]}_{+},\;\tau ,\zeta >0, \end{array}$$ (11)

In Equation 11, [*z*]_+_ = max{*z*, 0}. This expression allows the outside option to expand not only because of platform asymmetry, but also because of bargaining imbalance and excessive average prices. Thus market coverage becomes endogenous to the structural environment.

The indifferent consumer $\hat{x}$ is implicitly determined, as detailed in Equation 12


$$\begin{array}{l} \Delta U\left(x\right)\equiv U_{1}\left(x\right)-U_{2}\left(x\right)=0. \end{array}$$ (12)

Substituting Equations 6, 10, we obtain Equation 13


$$\begin{array}{l} 0=\left(\beta_{1}e_{1}-\beta_{2}e_{2}\right)-\left(\alpha_{1}p_{1}-\alpha_{2}p_{2}\right)+\left(\vartheta_{1}t_{1}-\vartheta_{2}t_{2}\right)\\ \;-\eta_{1}\frac{\left(t-t_{1}\right)x}{1+\kappa t_{1}}+\eta_{2}\frac{\left(t-t_{2}\right)\left(1-x-x_{0}\right)}{1+\kappa t_{2}}-\frac{\chi_{1}}{2}{\left(\frac{\left(t-t_{1}\right)x}{1+\kappa t_{1}}\right)}^{2}\\ \;+\frac{\chi_{2}}{2}{\left(\frac{\left(t-t_{2}\right)\left(1-x-x_{0}\right)}{1+\kappa t_{2}}\right)}^{2}. \end{array}$$ (13)

Accordingly, the demand allocation is as shown in Equation 14


$$\begin{array}{l} q_{1}=\hat{x},\;q_{2}=1-\hat{x}-x_{0},\;q_{0}=x_{0}, \end{array}$$ (14)

with *q*_0_ denoting the outside-option demand. Relative to the simpler linear formulation, this structure makes demand more sensitive to the interaction among recommendation intensity, market asymmetry, and non-linear mismatch costs. The outside-option design is introduced to capture a practically important feature of multimodal systems: when integration becomes too costly, too asymmetric, or insufficiently aligned with users' needs, some participants may rationally disengage from the focal system. Thus, the outside option is not merely a technical convenience; it reflects the possibility that poor alignment or excessive asymmetry reduces effective participation.

### 3.6 Cost structure of information utilization

Information utilization requires data collection, profiling, algorithmic computation, system deployment, and compliance effort. To capture both increasing marginal cost and the possibility that aggressive recommendation becomes disproportionately expensive in asymmetric environments, we define the information-cost function as Equation 15


$$\begin{array}{l} C_{i}\left(t_{i};\gamma ,\delta \right)=\frac{\lambda }{2}t_{i}^{2}+\frac{\mu }{3}t_{i}^{3}+\frac{\nu }{2}At_{i}^{2},\;\lambda ,\mu ,\nu >0, \end{array}$$ (15)

where A is the asymmetry index in Equation 3. Hence


$$\begin{array}{l} \frac{\partial C_{i}}{\partial t_{i}}=\lambda t_{i}+\mu t_{i}^{2}+\nu At_{i},\;\frac{\partial^{2}C_{i}}{\partial t_{i}^{2}}=\lambda +2\mu t_{i}+\nu A>0. \end{array}$$ (16)

The Equation 16 strengthens the model in two ways. First, the cubic term captures the fact that high-intensity information use may become sharply more expensive. Second, the asymmetry-adjustment term links cost directly to market structure, so that information strategies become harder to sustain when the environment is highly unbalanced.

### 3.7 Platform extraction and merchant residual payoffs

Platforms earn revenue through transaction commissions. Let ρ_*i*_ ∈ (0, 1) denote the base commission parameter. The effective commission rates are defined as Equation 17


$$\begin{array}{l} r_{1}=\frac{\rho_{1}\gamma }{\delta },\;r_{2}=\frac{\rho_{2}\left(1-\gamma \right)}{1-\delta }, \end{array}$$ (17)

implying that platform strength increases extraction capacity while merchant bargaining power constrains it (Wang et al., 2017).

Platform profits are therefore


$$\begin{array}{l} \pi_{i}=r_{i}p_{i}q_{i}-C_{i}\left(t_{i};\gamma ,\delta \right)-\sigma_{i}q_{i}^{2},\;i=1,2, \end{array}$$ (18)

In Equation 18, $\sigma_{i}q_{i}^{2}$ captures congestion, coordination, or service frictions associated with handling larger transaction volumes. This term allows information utilization to generate both expansion benefits and congestion costs.

Merchants retain the residual transaction revenue is shown in Equation 19:


$$\begin{array}{l} \pi_{Mi}=\left(1-r_{i}\right)p_{i}q_{i}+\omega_{i}e_{i}q_{i}-\frac{\varpi_{i}}{2}q_{i}^{2},\;i=1,2, \end{array}$$ (19)

where ω_*i*_*e*_*i*_*q*_*i*_ reflects the merchant-side gain from platform-provided service quality and $\frac{\varpi_{i}}{2}q_{i}^{2}$ captures increasing operating pressure when quantity expands. In this way, the model explicitly links platform-side information strategy to merchant-side payoff through both demand expansion and surplus redistribution.

### 3.8 Optimization problems and equilibrium conditions

Given platform choices in Stage 1, merchants choose prices in Stage 2 by solving, as shown in Equation 20


$$\begin{array}{l} \underset{p_{1}}{\max}\;\pi_{M1}\left(p_{1},p_{2};t_{1},t_{2},\rho_{1},\rho_{2}\right),\;\underset{p_{2}}{\max}\;\pi_{M2}\left(p_{1},p_{2};t_{1},t_{2},\rho_{1},\rho_{2}\right), \end{array}$$ (20)

As shown in Equation 21, with first-order conditions


$$\begin{array}{l} \frac{\partial \pi_{M1}}{\partial p_{1}}=0,\;\frac{\partial \pi_{M2}}{\partial p_{2}}=0. \end{array}$$ (21)

Anticipating these responses, platforms solve, as shown in Equation 22


$$\begin{array}{l} \underset{\rho_{1},t_{1}}{\max}\;\pi_{1}\left(\rho_{1},t_{1};\rho_{2},t_{2}\right),\;\underset{\rho_{2},t_{2}}{\max}\;\pi_{2}\left(\rho_{1},t_{1};\rho_{2},t_{2}\right), \end{array}$$ (22)

subject to merchant participation and consumer demand consistency. The interior first-order conditions are


$$\begin{array}{l} \frac{\partial \pi_{1}}{\partial \rho_{1}}=0,\;\frac{\partial \pi_{1}}{\partial t_{1}}=0,\;\frac{\partial \pi_{2}}{\partial \rho_{2}}=0,\;\frac{\partial \pi_{2}}{\partial t_{2}}=0. \end{array}$$ (23)

According to Equation 23, anticipating the merchants' optimal pricing responses, the two platforms make their decisions in Stage 1 by jointly choosing commission parameters and information-utilization intensities. Each platform evaluates the trade-off between the demand-expansion effect generated by better matching and the rising cost of information utilization under asymmetric market conditions. The equilibrium of the model is therefore defined as a sub-game-perfect equilibrium obtained by backward induction: merchants choose profit-maximizing prices conditional on platform strategies, platforms choose profit-maximizing commission and information policies while anticipating downstream responses, and the final allocation of demand across the two platforms and the outside option is consistent with consumer utility maximization. This equilibrium concept ensures that the model captures not only the direct value of information utilization, but also its strategic consequences after the reactions of all market participants are taken into account.

## 4 Results

This section evaluates the explanatory power of the proposed framework by examining whether it can generate coherent and interpretable outcome patterns across heterogeneous environments. The purpose is not merely to present isolated equilibrium statements, but to show how the interaction among information-utilization intensity, structural asymmetry, integration cost, and demand response leads to systematically different strategic regimes. In line with the methodology, the results are organized around four questions: What is the global structure of the strategy space? How do competitive asymmetry and information utilization reshape payoff trajectories? How is total welfare redistributed across environments? How sensitive are these outcomes to changes in information-processing cost and structural asymmetry?

To make the analytical narrative more transparent, this section combines conceptual summary tables with comparative synthesis figures. Rather than relying only on algebraic equilibrium statements, the section emphasizes strategic interpretation, cross-environment comparison, and distributional consequences. This structure makes it possible to show not only whether information utilization is adopted, but also why its role changes from redundancy, to defensive necessity, to asymmetry-reinforcing strategic leverage.

### 4.1 Strategy structure and analytical objective

The model allows each side to choose whether and to what extent it adopts enhanced information utilization. This produces three broad strategy profiles: NN, DN, and DD. However, these profiles do not represent isolated scenarios; rather, they form a structured progression across environments. Table 2 summarizes their strategic interpretation.

**Table 2:** Strategic interpretation of the three information-utilization profiles.

Strategy	Information structure	Expected market effect	Interpretation
NN	Neither side adopts enhanced information utilization.	Matching remains at the baseline level; allocation is governed mainly by structural symmetry and product substitutability.	Benchmark case used to assess whether additional information creates meaningful strategic value.
DN	Only one side adopts enhanced information utilization.	The adopting side improves matching and temporarily expands its market reach; the non-adopting side faces defensive pressure.	Transitional configuration revealing the short-run advantage of unilateral information utilization.
DD	Both sides adopt enhanced information utilization.	Matching quality improves on both sides, but the gains are redistributed according to asymmetry, cost, and downstream response.	Stable mutual-adoption outcome in intermediate environments; may remain symmetric or become structurally differentiated.

The analytical objective of this section is therefore not only to compare NN, DN, and DD mechanically, but to identify how the framework selects among them when the environment changes. In particular, the results below show that information utilization is neither uniformly optimal nor uniformly harmful. Instead, it becomes redundant in some environments, strategically unavoidable in others, and dominance-reinforcing in still others. To further address presentation clarity, the results are reported not only through equilibrium propositions but also through structured comparative tables and visual summaries. This combined presentation makes the cross-environment logic easier to follow and helps distinguish three different analytical questions: whether information utilization is adopted, what strategic role it plays after adoption, and how its gains are redistributed across actors.

### 4.2 Global strategic structure across heterogeneous environments

**Figure 2:**
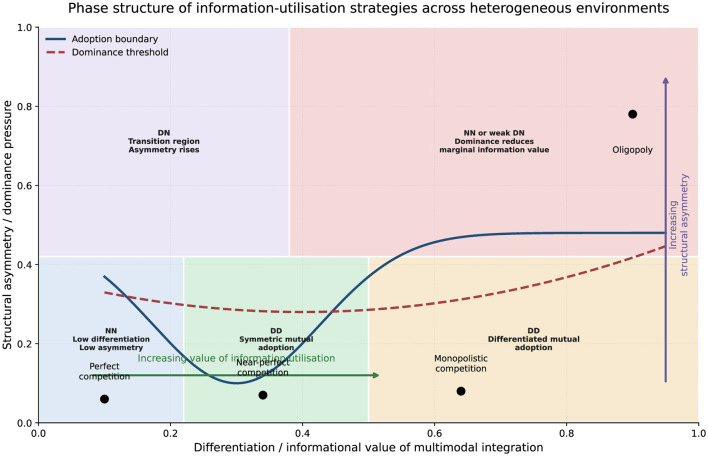
Strategy phase diagram across heterogeneous environments. The horizontal axis represents the increasing strategic value of information utilization associated with stronger differentiation and richer matching opportunities, whereas the vertical axis reflects the degree of structural asymmetry. The phase boundaries indicate that the equilibrium information-utilization profile is non-monotonic: non-adoption dominates in highly homogeneous environments, bilateral adoption emerges in intermediate regions, and information utilization becomes strategically differentiated or partially redundant as asymmetry increases.

The first major result of the framework is that the equilibrium strategy space is non-monotonic. Information utilization does not simply increase as the potential value of richer information grows. Rather, the equilibrium form depends on the joint movement of differentiation and structural asymmetry. Figure 2 summarizes this logic using a strategy phase diagram.

Figure 2 makes the comparative logic of the framework visually transparent. It shows that the equilibrium strategy profile does not evolve linearly with the informational richness of the environment. Instead, the strategic role of information utilization depends jointly on differentiation and asymmetry. In particular, the figure clarifies why bilateral information utilization is most likely to emerge in intermediate environments, whereas both highly homogeneous and highly dominated environments weaken the marginal incentive for additional information processing.

More specifically, four broad regions can be distinguished. First, when the environment is highly homogeneous and asymmetry is low, recommendation provides insufficient marginal value relative to cost, and the equilibrium remains NN. Second, once differentiation becomes positive but symmetry is largely preserved, unilateral adoption generates a temporary advantage, which pushes the system toward mutual adoption; the long-run outcome is DD with broadly uniform strategic behavior. Third, under stronger asymmetry and moderate-to-high informational value, both sides may still adopt information utilization, but the resulting DD equilibrium becomes internally differentiated: the stronger side captures a larger share of the benefit and increasingly relies on more aggressive extraction or differentiated response. Fourth, when asymmetry becomes very large, the incremental gain from additional information utilization falls because structural dominance itself already secures the position of the leading side. The equilibrium then shifts away from active bilateral information competition and tends back toward non-adoption or only weak unilateral deployment.

This result is methodologically important because it confirms that the framework does not mechanically treat information utilization as a universally desirable innovation. Instead, the same information mechanism can be irrelevant, unavoidable, or strategically excessive depending on environmental structure.

### 4.3 Environmental segmentation and comparative interpretation

To make the comparative logic explicit, the environments considered in the model are classified according to differentiation, platform asymmetry, and bargaining asymmetry. Table 3 summarizes this classification.

**Table 3:** Environmental classification used in the comparative analysis.

Environment	Differentiation level	Platform asymmetry	Merchant bargaining asymmetry
Perfect competition	Products are almost fully substitutable.	Platforms are symmetric.	Bargaining positions are balanced.
Near-perfect competition	Differentiation is positive but still limited.	Platforms remain broadly symmetric.	Bargaining positions remain balanced.
Monopolistic competition	Differentiation becomes moderate and strategically meaningful.	One side becomes stronger than the other.	Bargaining positions become uneven.
Oligopoly	Differentiation is high and the market becomes highly concentrated.	One side dominates the market.	Bargaining becomes highly dependent on the dominant side.

This segmentation is analytically useful because it links the structural parameters of the model to interpretable comparative regimes. The framework can therefore be read not as a collection of disconnected cases, but as a sequence of environments through which the strategic value of information utilization changes systematically.

### 4.4 Perfect competition: boundary validity

The first benchmark is the perfectly competitive case. Here, products are almost fully substitutable, the two sides are symmetric, and bargaining power is balanced. Under these conditions, enhanced information utilization creates little strategic advantage because there is almost no baseline mismatch to reduce. Recommendation therefore adds cost without generating sufficient allocative improvement. The equilibrium remains NN.

This result matters because it confirms the boundary validity of the framework. A model that always predicts active information use would fail to distinguish between environments in which richer processing is meaningful and those in which it is economically redundant. In the present framework, non-adoption arises endogenously when the scope for mismatch reduction is too limited to justify the cost of information utilization.

### 4.5 Near-perfect competition: strategic escalation under symmetry

The second benchmark concerns near-perfect competition. In this environment, differentiation is no longer zero, but asymmetry remains limited. The structural importance of this case is that a small positive mismatch creates room for information-based strategic escalation. A unilateral move toward enhanced information utilization temporarily improves matching and demand capture, which in turn pressures the rival side to respond. Table 4 summarizes the comparative outcomes.

**Table 4:** Comparative outcomes under near-perfect competition.

Outcome dimension	NN	DN	DD
Information utilization	None	One side adopts	Both sides adopt
Market allocation	Balanced but baseline matching quality	Temporary advantage for the adopting side	Returns to broad symmetry after mutual adoption
Pricing pattern	Uniform and restrained	Defensive reaction by the non-adopting side	Uniform pricing on both sides
Platform profit	Stable but limited	Short-run gain for the adopter, pressure on the rival	Mutual adoption erodes unilateral advantage
Merchant profit	Stable and broadly symmetric	Becomes more uneven in the transition phase	Returns to relative balance under bilateral adoption
Strategic interpretation	Benchmark equilibrium	Escalation phase	Prisoner's-dilemma type stable outcome

The result is a familiar but important one: near-perfect competition tends to push the system from NN toward DD, even if the joint-profit benchmark under NN is not necessarily worse. In other words, mutual information utilization emerges not because it is jointly optimal in a static sense, but because unilateral non-adoption becomes strategically unstable once one side moves first.

To make this strategic transition more explicit, Table 5 reports the payoff logic of the bilateral choice problem under symmetric competitive pressure.

**Table 5:** Payoff matrix of information-utilization strategies under symmetric competitive pressure.

Platform 1/Platform 2	Non-use	Use
Non-use	**NN strategy** Profits remain broadly balanced	**ND strategy** Platform 2 obtains the highest profit, while Platform 1 is competitively squeezed
**Use**	**DN strategy** Platform 1 obtains the highest profit, while Platform 2 is competitively squeezed	**DD strategy** Profits return to a relatively balanced distribution

NN strategy, neither side adopts enhanced information utilization; DN or ND strategy, only one side adopts enhanced information utilization; DD strategy, both sides adopt enhanced information utilization.

Table 5 shows why near-perfect competition has a strong escalation tendency. If one side adopts while the other does not, the adopter temporarily captures the highest payoff and pushes the rival into a weakened position. However, once the rival responds, the resulting DD configuration largely removes the unilateral advantage and restores a more balanced profit structure. The strategic implication is clear: unilateral adoption is individually attractive, but mutual adoption becomes the stable outcome because remaining at NN is not robust to deviation.

In a near-perfectly competitive environment, the equilibrium tends toward bilateral information utilization, and the resulting strategic pattern remains broadly uniform across the two sides.

Thus, the framework captures a transition from symmetry without information to symmetry with information. The result is analytically valuable because it demonstrates that information utilization can become strategically necessary even when it compresses the unilateral advantage that initially motivated adoption.

### 4.6 Monopolistic competition: differentiated information utilization under asymmetry

The most informative environment is monopolistic competition, because it is here that the framework must explain why information utilization no longer has the same meaning for both sides. Once asymmetry becomes more pronounced, the stronger side can use information utilization not only to improve matching but also to reinforce an already favorable position. The weaker side may still adopt information utilization, but mainly to reduce disadvantage rather than to dominate. Table 6 summarizes the comparative outcomes.

**Table 6:** Equilibrium comparison under monopolistic competition.

Outcome dimension	NN	DN	DD
Information utilization	None	Strong side adopts first	Both sides adopt
Relative market share	The stronger side already leads	The stronger side expands further	The stronger side remains dominant
Pricing pattern	Both sides mainly follow uniform behavior	Strong side becomes more aggressive; weak side reacts defensively	Strong side adopts differentiated response, weak side remains more uniform
Platform profit	Strong side leads, weak side survives	Strong side gains sharply, weak side is compressed	Both remain active, but the profit gap widens
Merchant profit	Uneven but positive on both sides	Merchant linked to the weak side becomes more vulnerable	Merchant outcomes remain asymmetric but stable
Strategic implication	Asymmetric coexistence	Dominance expansion	Stable differentiated equilibrium

This result shows that the framework can distinguish between *adoption* and *the strategic role of adoption*. Under monopolistic competition, DD may still emerge, but it no longer means symmetric mutual upgrading. Instead, the stronger side treats information utilization as an offensive instrument, while the weaker side treats it as a defensive instrument. The same formal strategy category therefore carries different strategic content depending on structural position.

To clarify this asymmetry more directly, Table 7 presents the corresponding payoff matrix under the second competitive setting.

**Table 7:** Payoff matrix of information-utilization strategies under asymmetric competitive pressure.

Platform 1/Platform 2	Non-use	Use
**Non-use**	**NN strategy** Neither side achieves profit maximization	**ND strategy** Platform 1 receives the lower profit, whereas Platform 2 receives the higher profit
**Use**	**DN strategy** Platform 1 receives the higher profit, whereas Platform 2 receives the lower profit	**DD strategy** Both sides improve profits, but the gain remains structurally uneven

NN strategy, neither side adopts enhanced information utilization; DN or ND strategy, only one side adopts enhanced information utilization; DD strategy, both sides adopt enhanced information utilization.

Table 7 indicates that, unlike the near-perfectly competitive case, the NN outcome is no longer attractive for either side. Bilateral information utilization improves profits relative to non-adoption, but the two sides do not benefit symmetrically. Unilateral adoption still generates a large payoff gap, and even under DD the stronger side remains in a structurally superior position. This is precisely why the monopolistic-competition equilibrium is best understood as a differentiated DD regime rather than as a symmetric mutual-upgrading outcome.

In a monopolistic competitive environment, both sides may adopt enhanced information utilization, but the strong side captures a disproportionate share of the gain and exhibits more differentiated strategic behavior than the weak side.

This is one of the clearest advantages of the proposed framework. Simpler models often treat information utilization as either purely efficiency-enhancing or purely competition-intensifying. Here, the same mechanism can improve matching while simultaneously widening the distribution of gains.

### 4.7 Oligopoly: redundancy of information utilization under dominance

The final benchmark is the oligopolistic environment. In this case, structural asymmetry is so strong that one side already occupies a highly dominant position. The framework predicts that under such conditions the marginal strategic return to additional information utilization declines sharply. Recommendation is no longer necessary to secure dominance, because market concentration itself already performs that function. Table 8 summarizes the logic.

**Table 8:** Outcome summary under oligopoly.

Outcome dimension	No enhanced utilization	Enhanced utilization
Market share	The dominant side already serves nearly the entire covered market	Market coverage changes little because dominance is already secured
Pricing	High and structurally sustained	Shows only limited additional adjustment
Platform benefit	Gains arise mainly from structural dominance	Additional information generates little incremental return
Merchant position	Merchants remain dependent on the dominant side	Their bargaining position does not improve materially
Strategic implication	Recommendation is unnecessary	Recommendation becomes redundant rather than expansionary

This result again strengthens the boundary validity of the framework. Information utilization is not modeled as an intrinsically beneficial action. Its return depends on whether it changes the competitive constraint faced by the dominant side. In oligopoly, that constraint is already weak, so the need for additional information-based competition falls correspondingly.

In highly concentrated environments, enhanced information utilization becomes strategically redundant because structural dominance already secures most of the attainable market advantage.

### 4.8 Profit reallocation across the environmental sequence

**Figure 3:**
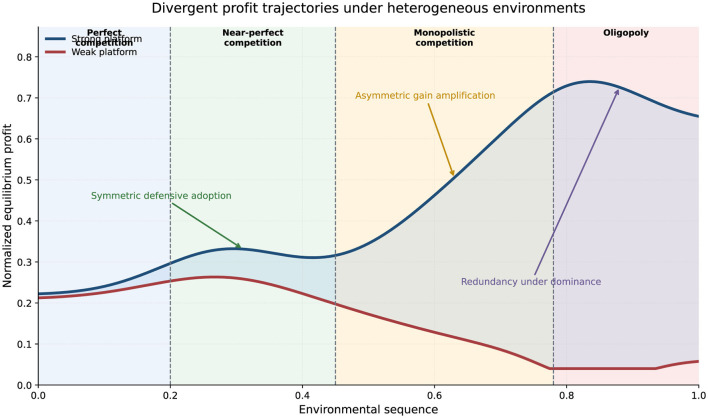
Divergent equilibrium profit trajectories across heterogeneous environments. The curves report normalized profit levels for the strong and weak sides as the market moves from perfect competition to oligopoly. The figure shows that information utilization generates limited relative advantage under symmetric competition, but substantially amplifies profit divergence once structural asymmetry becomes more pronounced. In highly concentrated environments, the incremental value of further information utilization declines because dominance itself becomes the primary source of profit.

The comparative synthesis figures make the cross-environment pattern more explicit. Figure 3 reports the normalized profit trajectories of the strong and weak sides across the full environmental sequence.

Figure 3 further illustrates that the consequences of information utilization are strongly distributional. In the early part of the environmental sequence, both sides remain relatively close in profit because competitive pressure limits the extent to which one side can internalize the gain from better matching. Once the market enters a more asymmetric regime, however, the stronger side captures a disproportionate share of the benefit, and the gap widens rapidly. The figure therefore supports the view that information utilization changes not only market efficiency, but also the balance of strategic advantage.

Two observations stand out. First, under perfect and near-perfect competition, the profit gap remains relatively limited. Even when both sides adopt information utilization, competition forces them into a defensive equilibrium in which the unilateral advantage of early adoption is not fully retained. Second, once asymmetry becomes more pronounced, the profit paths diverge sharply. The stronger side captures a rising share of the total gain from information utilization, while the weaker side faces a declining relative return.

Figure 3 therefore confirms that information utilization is not only a matching device but also a redistribution mechanism. It alters not just efficiency, but the balance of gains between structurally unequal sides.

The originally drafted conceptual summary figure is retained here as a complementary illustration of strategic evolution.

**Figure 4:**
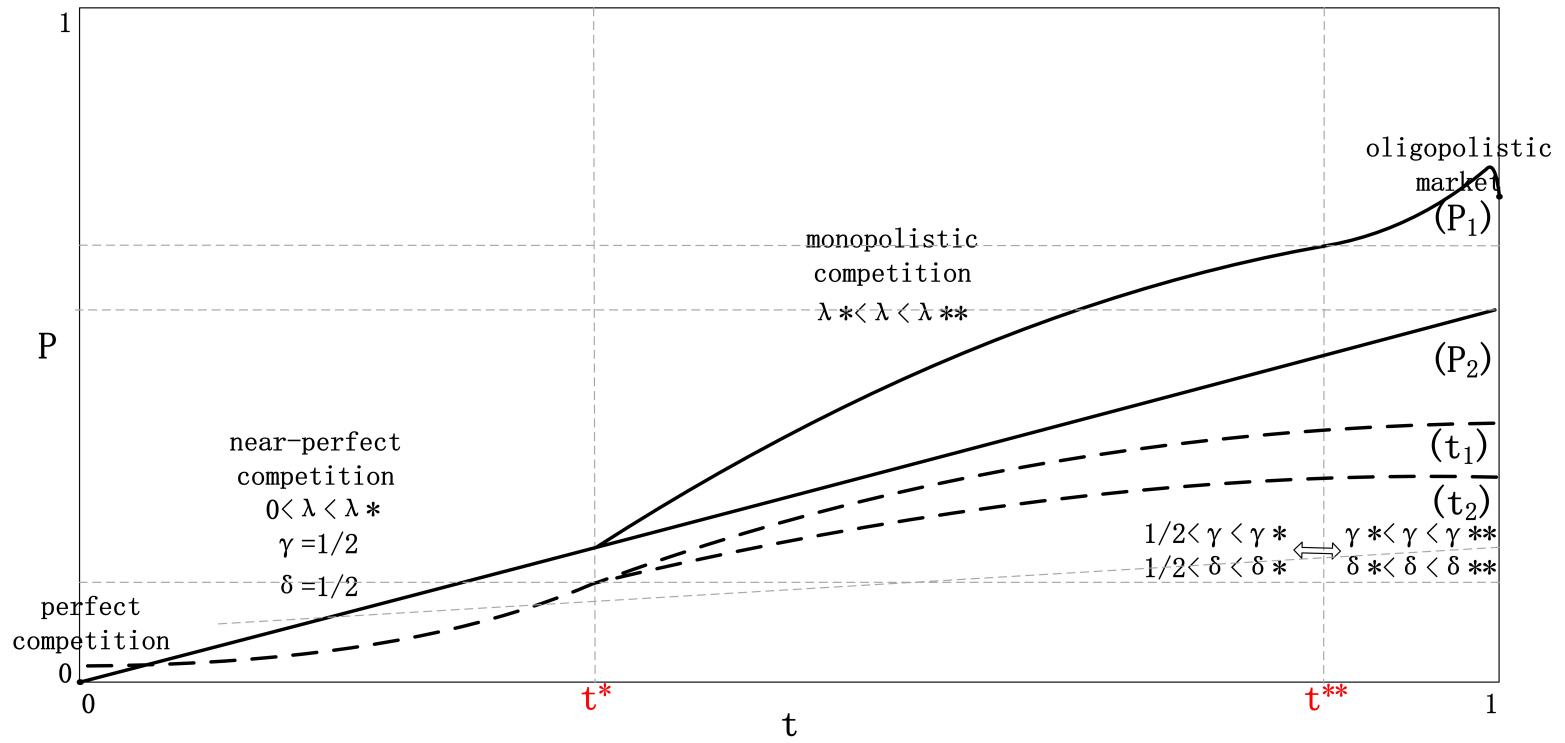
Summary of information utilization and pricing strategies.

Figure 4 complements the profit curves by showing the qualitative co-movement of information adoption and pricing stance. Read together, Figures 3, 4 indicate that the transition from symmetric to asymmetric environments involves not only greater adoption intensity, but also a progressive shift from defensive coordination to advantage-preserving strategic differentiation.

### 4.9 Welfare decomposition and distribution of gains

**Figure 5:**
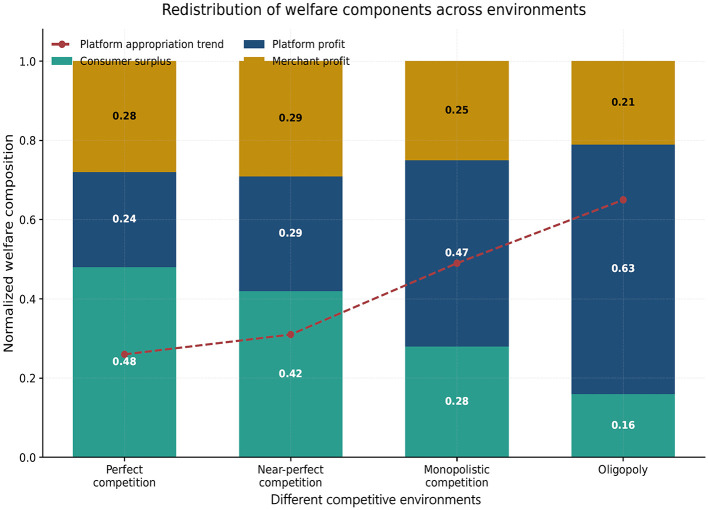
Welfare decomposition under alternative environments. The stacked bars show the relative contributions of consumer surplus, platform profit, and merchant profit to total normalized welfare. The figure indicates that welfare is comparatively balanced under symmetric competition, shifts toward platform appropriation under monopolistic competition, and becomes increasingly concentrated in platform profit under oligopoly. This pattern suggests that the consequences of information utilization depend not only on efficiency gains, but also on how those gains are redistributed across market participants.

A further advantage of the framework is that it distinguishes between total gains and their composition. Figure 5 decomposes normalized welfare into consumer surplus, platform profit, and merchant profit across the four benchmark environments.

Figure 5 shows that total welfare and welfare composition should be distinguished analytically. Even when information utilization improves matching quality, the gains are not distributed uniformly across participants. Under relatively symmetric environments, the welfare structure remains balanced, but as asymmetry increases, a growing share of welfare is transferred toward platform profit. This result helps explain why information-rich systems may appear efficient while still producing increasingly uneven distributive outcomes.

The welfare pattern is not uniform. Under perfect competition, the welfare composition is relatively balanced, with consumer surplus occupying a comparatively larger share. In near-perfect competition, bilateral information utilization can improve matching and temporarily support consumer-side gains, but competitive escalation compresses the extent to which those gains are transformed into durable profit advantages. In monopolistic competition, the composition shifts toward the stronger side: platform profit rises as a share of total welfare, while merchant profit and consumer surplus become relatively compressed. Under oligopoly, the concentration of welfare in platform profit is strongest, indicating that dominance rather than recommendation becomes the main driver of surplus appropriation.

This decomposition is important because it shows that the framework is not limited to predicting strategy choice. It also helps explain how the gains from information utilization are redistributed among the participating groups.

The original profit-change figure can still be retained as a legacy summary graphic.

**Figure 6:**
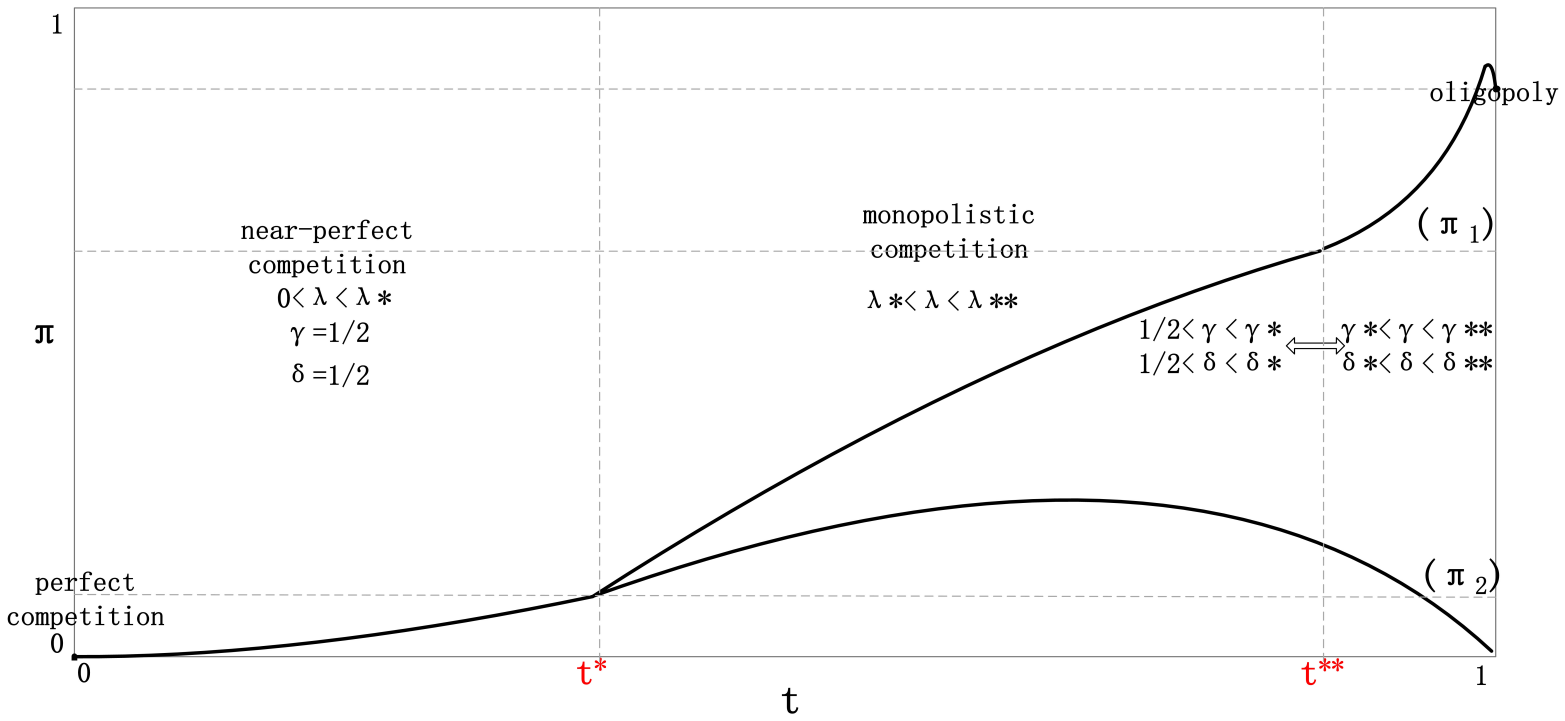
Profit change curves for both platforms.

Figure 6 serves as a visual bridge between the original conceptual presentation and the richer welfare decomposition. It reinforces the idea that changes in platform payoff should be interpreted jointly with shifts in consumer and merchant outcomes, rather than as an isolated indicator of system improvement.

### 4.10 Sensitivity to cost and structural asymmetry

**Figure 7:**
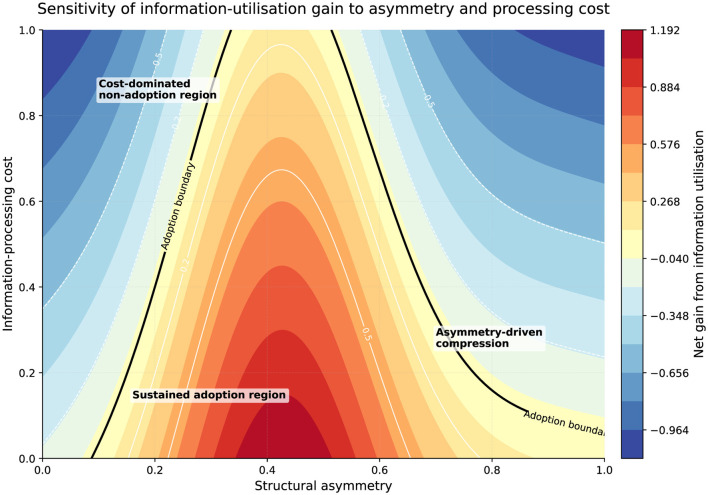
Sensitivity of the net gain from information utilization to information-processing cost and structural asymmetry. Warmer regions indicate a higher net strategic return to information utilization, whereas cooler regions indicate that the marginal gain is weak or negative. The contour line marks the approximate adoption boundary. The figure shows that information utilization is most sustainable when cost remains moderate and asymmetry is neither too low nor too high, while excessive cost or excessive dominance both reduce the equilibrium incentive to adopt.

The final result concerns robustness. Figure 7 maps the net gain from information utilization over the joint space of information-processing cost and structural asymmetry.

Figure 7 provides a compact robustness check for the main mechanism of the model. The figure confirms that the gain from information utilization is bounded by a clear adoption region rather than increasing without limit. Moderate asymmetry and manageable processing cost create the most favorable conditions for adoption, whereas very high asymmetry or very high cost both shrink the feasible region. This supports the argument that information utilization is a conditional strategic response rather than a universally dominant choice.

The figure shows a clear adoption boundary. When cost is low and asymmetry is moderate, the net gain from information utilization is positive, so adoption is sustained. This corresponds to the intermediate regions identified earlier, where recommendation helps reduce mismatch and remains strategically relevant. By contrast, when processing cost becomes high or asymmetry becomes too strong, the gain turns negative. Recommendation then becomes either unattractive or only weakly sustained, because its marginal benefit is dominated by cost or by pre-existing structural advantage.

This result strengthens the framework in two respects. First, it confirms that the conclusions are not driven by a single parameter value, but hold across a continuous parameter region. Second, it shows that asymmetry has a dual effect: moderate asymmetry can increase the strategic reward to information utilization for the stronger side, but excessive asymmetry eventually reduces the need for information-based competition because dominance itself substitutes for it.

### 4.11 Cross-environment comparative interpretation

Taken together, the results demonstrate that the proposed framework has three forms of explanatory strength.

First, it has strong *boundary validity*. The framework recovers intuitive outcomes in the extreme cases: recommendation is not adopted when the environment is too homogeneous, and it becomes redundant again when dominance is already sufficiently strong.

Second, it has strong *transition validity*. Between these extremes, the framework identifies a nontrivial strategic path: from non-adoption, to bilateral defensive adoption, to differentiated bilateral adoption under asymmetry, and finally to strategic redundancy under highly concentrated environments.

Third, it has strong *structural interpretability*. Each equilibrium pattern can be traced back to the interaction among mismatch reduction, outside-option expansion, information cost, bargaining asymmetry, and the distribution of strategic power. In this sense, the model does not merely fit isolated scenarios; it offers a coherent analytical lens for understanding why information utilization may appear indispensable in some environments, defensive in others, and unnecessary in yet others.

Overall, the findings suggest that information utilization should not be evaluated as a universally beneficial mechanism. Its value is conditional, environment-dependent, and closely tied to how structural asymmetry reshapes both competition and the distribution of gains.

## 5 Conclusion

This study develops a conceptual analytical framework for understanding how multimodal information utilization evolves across heterogeneous processing environments in neuroscience and perception science. Rather than assuming that the integration of additional modalities is always beneficial, the framework treats information utilization as a conditional strategic response whose value depends on the interaction among baseline differentiation, structural asymmetry, bargaining conditions, and information-processing cost. In this way, the study shifts the discussion from a purely technical question of whether more modalities improve performance to a broader analytical question of when, for whom, and under what conditions enhanced information utilization produces meaningful system-level gains.

Several limitations should also be acknowledged. First, the present study is conceptual and analytical rather than empirical, so its conclusions should be interpreted as theoretically grounded comparative propositions rather than direct empirical estimates. Second, the framework abstracts away from many complexities that characterize real multimodal systems, including dynamic adaptation over time, missing-modality mechanisms, multi-agent coordination, and task-specific differences in interpretability or reliability. Third, the current model emphasizes strategic and structural heterogeneity, but does not explicitly incorporate learning dynamics, regulatory constraints, or ethical safeguards, all of which are increasingly important in real neuroscience and perception-related applications.

## Data Availability

The original contributions presented in the study are included in the article/supplementary material, further inquiries can be directed to the corresponding author.
